# Judicial and legislative practice and related suggestions on off-label drug use in China

**DOI:** 10.1186/s12913-023-09293-y

**Published:** 2023-03-30

**Authors:** Wenjie Si, Panpan Ma

**Affiliations:** Department of International Medical Services, Peking Union Medical College Hospital, Beijing, China

**Keywords:** Off-label drug use, Legal liability, Precedents of jurisdictions

## Abstract

**Background:**

Off-label drug use exists widely in medical practice and is also an area which easily triggers controversy between patients and medical institutions. Previous studies have identified the reasons why off-label drug use long exists. However, there is no multidimensional analysis on real judicial precedents about off-label drug use. This study aimed to investigate the dispute points on off-label drug use based on real cases in China, and proposed suggestions based on newly-leased Physicians Law.

**Methods:**

Our study is a retrospective study with all the 35 judicial precedents on off-label drug use extracted from China Judgments Online from 2014 to 2019. This study mainly used the methods of statistical analysis, inferential analysis, exemplification, literature summarization and comparative analysis.

**Results:**

According to the analysis of the 35 precedents of jurisdictions from 11 different aspects, it can be seen that the second-instance and retrial rates of this kind of cases are high, and the disputes between patients and medical institutions are fierce. In judicial practice of off-label drug use, medical institutions are determined whether to bear civil liability according to the constituent elements of medical tort liability: the rate of medical institutions’ bearing liability for off-label drug use is not high, and medical institutions are not directly identified as infringing acts and they don’t bear tort liability. The clear provisions about off-label drug use in Law of the People’s Republic of China on Physicians which was implemented in March 2022 confirm this at the legislative level.

**Conclusions:**

By analyzing the current judicial practice of China’s off-label drug use cases, and summarizing the dispute points between medical institution and patients, the constituent elements of tort liability, and the rules of evidence etc., suggestions are proposed to further regulate off-label drug use and promote safe and rational drug use.

## Background

Off-label drug use (OLDU) refers to the inconsistency of the patient’s age, indications, administration method, dose, and route of administration with the drug instruction provided by the manufacturer [1]. OLDU in medical institutions has its rationality and inevitability [2], because it can meet the needs of disease treatment to a certain degree, and promote the development of clinical drug use [3]. Therefore, OLDU exists widely in clinical practice at home and abroad [4]. Some research indicates that 21% of drugs worldwide are used off-label [5]. However, patients often firmly agree to the authority of drug instructions, and believe that the medical institutions’ failure to follow the instructions is a violation of the diagnosis and treatment norms, and this will result in disputes. No matter in theory and in practice, this controversy has always been concerned, but the current theoretical research mainly focuses on the investigation and recommendations of OLDU in a certain specialty field. Since 2014, there have been a large number of cases concerning OLDU in judicial practice in China. Meanwhile, relevant national and local legislative practices have also explored OLDU. The “Law of the People’s Republic of China on Physicians”, which was implemented on March 1, 2022, clearly stipulated OLDU from the legal level for the first time, providing a basic legal basis for this issue. However, in medical activities and judicial practice, there are still disputes between medical institutions and patients. This paper sorts out and analyzes the judicial precedents and legislation on OLDU in China, and on this basis provides relevant suggestions for medical institutions to regulate OLDU.

Before 2022, there was no specific legislation for OLDU in China. The “Drug Administration Law” clearly stipulates the accuracy of drug instructions and the strict verification of drug dispensers in medical institutions. Paragraph 9 of Article 6 of the “Prescription Administrative Policy” stipulates that drugs can be used in excess doses under special circumstances, but the reasons should be indicated and signed again. Although the law does not expressly prohibit the prescribing right of OLDU, there is no positive regulation either. Therefore, in clinical practice, there are inconsistencies about whether or not to use off-label drugs, the responsibility of using off-label drugs, under what circumstances can off-label drugs be used, and what procedures and rules need to be followed. In view of this, professional societies in various provinces and cities have also launched a series of consensuses from the perspective of clinical guidance. For example, the “Expert Consensus on Drugs’ Unregistered Use” launched by Guangdong Pharmaceutical Association in March 2010 is the first industry norms for OLDU issued by a professional association in China [6]. In July 2013, the Professional Committee of Clinical Pharmacy of Sichuan Pharmaceutical Association issued the “Expert Consensus on Off-label Drug Use of Sichuan Pharmaceutical Association (Discussion Draft)”. These consensuses do not have legal force, nor are they uniform norms across the whole country.

It is worth noting that 2022 Law of the People’s Republic of China on Physicians regulates OLDU for the first time, making China a country that legislates and regulates OLDU after the United States, France, Germany, the Netherlands, Italy, New Zealand, Japan, and India [7]. The Article 29 stipulates that “Physicians shall adhere to the principles of safe, effective, economical and rational drug use, and follow the guiding principles of clinical application, clinical diagnosis and treatment guidelines, and drug instructions when use drugs. Under special circumstances, such as the absence of effective or better treatment means, after obtaining the patient’s clear informed consent, a physician may use the drug that is not specified in the drug instructions but has evidence-based medical evidence for treatment. Medical institutions shall establish a management system to examine the suitability of physicians’ prescriptions and medication orders, and strictly regulate physicians’ medication behaviors.” This provision provides legislative safeguards for medical institutions and medical personnel to use off-label drugs. At the same time, restrictions are made: (1) Provided that there is no effective or better treatment means and under other special circumstances. (2) In terms of procedure, the physicians are required to obtain patients’ explicit informed consent. The Law of the People’s Republic of China on Physicians also requires that medical institutions should establish a management system to review the appropriateness of physicians’ prescriptions and medication orders, and strictly regulate physicians’ medication behavior.

When analyzing the lawsuits involving OLDU, this paper uses several terms. Here are their brief definitions: (1) Tort liability refers to the liability that arises as the result of one person’s causing property damage to another according to civil law. (2) First instance means the first court hearing. (3) Second instance means that when either party of a lawsuit refuses to accept the judgement of first instance, he can appellate to a higher court before the judgement takes legal effect. (4) A new trial in which issues already litigated and to which the court has already rendered a verdict or decision are reexamined by the same court. (5) The burden of proof means the duty of presenting a certain amount of evidence in order to meet the legal requirements for establishing the entitlement of the party in a case to the outcome sought.

## Methods

By searching precedents of jurisdictions published on China Judgments Online, an authority site run by the Supreme People’s Court of the People’s Republic of China, there were 35 lawsuits involving OLDU, and the time span of judgments was 2014–2019. This study analyzed these 35 cases from 11 different dimensions to investigate the current condition of juridical precedents caused by OLDU. The 35 cases were comprehensively analyzed from 11 different aspects, such as the cause of the cases, the trial procedure, trial results, distribution of involved medical institutions, involved drugs and departments, etc. When analyzing and discussing about these cases, this paper uses the method of inferential analysis; when reviewing China’s legislation on the regulation of OLDU, and proposing suggestions, this paper also uses the methods of exemplification, literature summarization and comparative analysis.

Since one dimension is to analyze the medical institutions involved in these 35 lawsuits, it is necessary to introduce the classification of Chinese medical institutions. Generally, according to whether they are owned and funded by the government, Chinese medical institutions can be divided into public and private hospitals. Public hospitals in China are organized according to a 3-tier system that recognizes a hospital’s ability to provide medical care, medical education, and conduct medical research. Based on this, public hospitals are designated as Primary, Secondary and Tertiary institutions (Tertiary is better than Primary). Further, based on the level of service provision, size, medical technology, medical equipment, and management and medical quality, these 3 grades are further subdivided into 3 subsidiary levels: A, B and C (A is better than C). This results in a total of 9 levels.

## Results

### Overall picture of judicial precedents on OLDU

Judging from the cause of the cases, there were mainly 34 cases of medical damage liability disputes, in addition to 1 case of medical service contract dispute. Judging from the trial procedure, there were 20 cases in the first instance, 14 cases in the second instance, and 1 case in the retrial (Table 1), reflecting that the lawsuit triggered by OLDU is likely to lead to the second instance, and the disputes and contradictions are more prominent. Judging from the subject of disputes, medical institutions are all defendants in the 20 cases of first instance. Among the 15 cases of second instance and retrial, 7 cases are applied by medical institutions, and 8 cases by patients, which states that in the litigation practice, medical institutions and patients have great difference and their dispute is very fierce, because they don’t accept the definition made by the court of first instance in terms of fact finding and legal liability. Judging from the trial results, in the second-instance and retrial cases, 14 cases were upheld, accounting for 93.33%, and only 1 case, accounting for 6.67%, was amended because the facts were unclear, indicating that the court system is relatively consistent in the understanding of the disputes and legal liability caused by OLDU. Judging from patient outcomes, among the 35 cases, 21 of the patients died, accounting for 60%, which reflects that when the patient dies or the condition worsens, the patient side is easily to question OLDU behavior of the medical institution, which results in medical disputes and lawsuits. Judging from the duration of precedents (Table 2), it took an average of 2.25 years per case from the occurrence of the case till the day when an effective judgment was made. Considering of the time and money cost by medical disputes and lawsuits triggered by OLDU, both patients and medical institutions suffer a lot from that.

**Table 1:** Trial Procedure of Precedents on OLDU

Closure of Trial	Quantity	Proportion
First instance	20	57.14%
Second instance	14	40.00%
Retrial	1	2.86%
Total	35	100%

**Table 2:** Lawsuit Duration of Precedents on OLDU

Lawsuit Duration	Quantity of Cases	Proportion
Within 1 year	2	5.71%
1≤ ~ <2 years	11	31.43%
2≤ ~ <3 years	6	17.14%
3≤ ~ <4 years	10	28.57%
4≤ ~ <5 years	4	11.43%
5 years	2	5.71%

### Distribution of medical institutions involved in precedents of jurisdictions

Among the 35 judicial precedents, there are 36 medical institutions involved, with the highest proportion of Class A tertiary hospitals, accounting for 72.2%, and higher-level medical institutions have more cases (Table 3). Judging from the occurrence area, Beijing, the capital city, has the most cases, which is 11, Guangdong Province (in South China) has 5 cases, and Zhejiang Province and Jiangsu Province (both in East China) have 4 cases each. China is divided into 5 areas geographically, East China, South China, Central China, West China and Northeast China. The economic condition in East China and South China is much better than that in the other three areas. Overall, the number of cases is positively correlated with regional economic development and medical resources (Table 4). Judging from the distribution of medical institutions involved in the precedents, it can be seen that the higher a hospital’s level is, and the stronger a hospital’s strength is, the physicians there are more willing to use OLDU.

**Table 3:** Basic Information of Medical Institutions Involved in Precedents of Jurisdictions on OLDU

Hospital Level	Quantity	Proportion
Class A Tertiary Hospital	26	72.2%
Class A Secondary Hospital	3	8.3%
Class B Secondary Hospital	4	11.1%
Primary Hospital	2	5.5%
Private Hospital	1	2.7%
Total	36	100%

**Table 4:** Distribution of Areas Involved in Precedents of Jurisdictions on OLDU

Province	Quantity	Quantity of Patients in 2019
Beijing Municipality (Capital City)	11	248,863,924
Guangdong Province (South China)	5	891,797,672
Zhejiang Province (East China)	4	681,331,526
Jiangsu Province (East China)	4	617,216,469
Anhui Province (Central China)	2	333,159,292
Henan Province (Central China)	2	610,202,911
Sichuan Province (West China)	2	560,264,451
Shanghai Municipality (East China)	1	275,599,946
Shanxi Province (East China)	1	131,456,549
Liaoning Province (Northeast China)	1	199,875,532
Jilin Province (Northeast China)	1	110,419,561
Hubei Province (Central China)	1	353,825,758

### Types, drugs and departments involved in precedents of jurisdictions on OLDU

It is generally believed that OLDU can be divided into off-indication drug use, over-dose and frequency drug use, over-administration route drug use, over-suitable group and age drug use, etc. [8] Among the 35 cases, off-indication drug use and over-dose and frequency drug use are the main types of disputes between medical institutions and patients (Table 5). One case may contain more than one type of OLDU, for example, one case may contain both off-indication drug use and over-dose drug use. Meanwhile, a total of 60 categories of drugs were involved (Table 6), and a total of 22 departments are involved. Except for the relatively concentrated disputes in Obstetrics, the distribution of other departments is relatively scattered (Table 7).

**Table 5:** Main Types and Proportion of OLDU Involved in Precedents of Jurisdictions

Types of OLDU	Quantity of Related Cases	Proportion
Off-indication Drug Use	23	65.71%
Over-dose and Frequency Drug Use	19	54.29%
Over-administration Route Drug Use	2	5.71%
Over-suitable Group and Age Drug Use	2	5.71%

**Table 6:** Drugs Involved in Precedents of Jurisdictions on OLDU

Levofloxacin and Sodium Chloride Injection	Torasemide Injection
Levocarnitine Injection	Sodium Bicarbonate Injection
Cefoxitin Sodium for Injection	Recombinant Human Erythropoietin Injection [Epiao](Prefilled Type)
Cefoperazone Sodium and Sulbactam Sodium for Injection[Sulperazon]	Alprostadil Injection
Sterile Water for Injection	Nikethamide Injection
Benzylpenicillin Sodium for Injection	Misoprostol Tablets
Bortezomib for Injection	Mifepristone Tablets
Pantoprazole sodium for Injection	Cinepazide Maleate Injection[Kelinao]
Piperacillin Sodium and Tazobactam Sodium for Injection	Potassium Chloride Injection
Methotrexate for Injection, Methotrexate Tablets	Gentamycin Sulfate Injection
Sodium Valproate for Injection, Sodium Valproate Oral Solution, Sodium Valproate Sustained-release Tablets	Clopidogrel Bisulfate Tablets[Plavix]
Hemocoagulase for Injection	Clopidogrel Hydrogen Sulphate Tablets
Esomeprazole Sodium for Injection	Atropine Sulfate Injection
Medium and Long Chain Fat Emulsion Injection (C6 ~ 24)[Lineng (Chinese Brand)]	Atropine Sulfate Eye Gel
Tirofiban Hydrochloride and Sodium Chloride Injection[Xinweining (Chinese Brand)]	Carbamazepine Tablets
Epinephrine Hydrochloride Injection	Metoprolol Tartrate Tablets
Diprophylline Tablets	Metronidazole and Sodium Chloride Injection
Propranolol Hydrochloride Tablets	Puerarin and Glucose Injection
Moxifloxacin Hydrochloride Tablets	Compound Amino Acid Injection
Moxifloxacin Hydrochloride and Sodium Chloride Injection [Avelox]	Fluorouracil Injection
Lidocaine Hydrochloride Injection	Low Molecular Weight Heparin Sodium Injection
Arginine Hydrochloride Injection	Bruceolic oil emulsion injection
Dopamine Hydrochloride Injection	Isosorbide Mononitrate Sustained Release Tablets [Imdur]
Ambroxol Hydrochloride Injection	Sulfotanshinone Sodium Injection
Esmolol hydrochloride injection [Ailuo (Chinese Brand)]	Atorvastatin Ccalcium Tablet [Lipitor]
Isosorbide Dinitrate Tablets	Aspirin Enteric-coated Tablets
Cimetidine Injection, Cimetidine Tablets	0.9% Sodium Chloride Injection

**Table 7:** Departments Involved in Precedents of Jurisdictions on OLDU

Department	Quantity of Cases
Obstetrics	9
Invasive Technology Department	3
Hematology	2
Cardiology	2
Neurology	2
Cerebral Surgery	2
Medical Cosmetology	1
Ophthalmology	1
Thoracic Surgery	1
Department of Digestive Diseases	1
Gastric Surgery	1
Nephrology	1
Dermatology	1
MICU	1
Internal Medicine (General Department)	1
Endocrinology	1
Anesthesiology Department	1
Geriatrics	1
Respiratory	1
Orthopedics Department	1
Hepatobiliary and Pancreatic Surgery	1
Pediatrics	1

### Determination of legal liability in the precedents of jurisdictions on OLDU

Disputes between Medical Institutions and Patients over Legal Liability for OLDU.

In judicial practice, whether OLDU constitutes tort liability is the focus of disputes between patients and medical institutions. Judging from the causes of action put forward by patients in judicial judgments, patients believed that OLDU existed in all cases (accounting for 100%). They believe that medical institutions should be responsible for OLDU are mainly concentrated on: (1) The instructions have been approved through legal procedures and are highly effective. OLDU violates diagnosis and treatment norms, and causes medication errors. (2) Medical institutions failed to fulfill their obligation to fully inform patients and violated patient’s right to know. In 20 cases, the patients or their families claimed that the medical institutions’ notification was not standardized, and they did not fulfill the obligation of special risk notification, violating patients’ rights to know and to choose. (3) Medical institutions had the behavior of modifying or concealing medical records. In 19 cases, the patients or their families claimed that the medical records of the medical institutions were incomplete, the behavior of OLDU was not recorded, the contents of the medical records were modified, relevant contents were concealed, the writing was incomplete, and there was no physician’s signature, etc. Medical institutions’ main reasons to refute are: (1) There are clinical indications to prove that OLDU is reasonable, and there are many supporting evidence, including domestic and foreign guidelines, foreign drug instructions, expert consensus, professional textbooks, or evidence-based medicine evidences, such as authoritative literature reports and case reports. (2) The development of patients’ condition is based on his own situation and has nothing to do with drug use behavior.

### Determination of legal liability in the precedents of jurisdictions on OLDU

The medical damage caused by OLDU is applicable to the constituent elements of general medical damage, namely: the medical institution has medical behaviors that violate the routine of diagnosis and treatment (generally based on judicial expertise); there are facts of damage, that is, the patients are harmed; there is a causal relationship between the medical behavior and the damage; the medical practice has fault or negligence. Among the 35 cases, judging from fact-finding, the court identified that OLDU in medical institutions constituted medical damage in 17 cases, did not constitute medical damage in 15 cases, and 3 cases were not clearly identified (Table 8). Judging from the degree of responsibility born by medical institutions, the cases of bearing full responsibility or primary responsibility are very few, and the cases of bearing equal responsibility, secondary responsibility and no responsibility are quite a lot (Table 9). Judging from the amount of compensation awarded, the total amount of compensation is 5,928,018.45 Yuan (around 888,017.16 US dollars), with an average of 174,353.48 Yuan (around 26,118.15 US dollars) per case, and a maximum compensation of 968,451.29 Yuan (around 45,074.00 US dollars). A total of 10 cases were judged not to be liable for compensation, accounting for 28.57%, and one case was voluntarily paid 10,000 Yuan (around 1,498 US dollars) compensation by a medical institution.

**Table 8:** Determination Condition of Legal Liability on OLDU

Determination Condition of Legal Liability	Quantity of Cases	Proportion
OLDU behavior is confirmed to bear liability	17	48.57%
OLDU behavior is confirmed not to bear liability	15	42.86%
There is no clear determination of liability	3	8.57%

**Table 9:** Determination of Legal Liability on OLDU

Liability of compensation	Quantity	Proportion
Full liability	2	5.71%
Primary liability	6	17.14%
Equal liability	7	20.00%
Secondary liability	6	17.14%
Minor liability	1	2.86%
No liability	13	37.14%

Although the 35 cases of judicial precedents analyzed in this paper occurred before the legislation, the judgments of judicial practice are consistent with the spirit of 2022 Law of the People’s Republic of China on Physicians.

### Analysis on legal theory of OLDU

According to the provisions of the Civil Code of China, medical damage liability can be divided into the liability for medical technology damage, the liability for medical ethics damage and the liability for medical product damage. OLDU refers that medical institutions and their medical personnel violate the usage of instruction, and fail to provide patients with “the required medical level”, resulting in personal or property damage to patients. The imputation principle of medical damage liability caused by OLDU belongs to principle of fault liability. However, from the cases’ analysis, it can be seen that patients are naturally in weak position in terms of burden of proof, including medical records stored in medical institutions and patients’ weakness in professional knowledge. The evidence provided by patients is often only the provisions of the medical record and the instructions. Therefore, in terms of the effectiveness of proof, it can only prove the existence of OLDU behavior, but as to whether the medical behavior causes damage, whether there is subjective negligence, and whether there is a causal relationship between the actual damage result and the behavior, it cannot meet the proof standards required for medical damage liability. It is suggested to introduce relief system of burden of proof, and transfer the burden to the defendant (i.e. medical institution or its medical personnel) on the basis of the patient’s initial performance of the burden of proof. This transfer can help to balance the medical institution-patient gap [9].

## Discussion

In daily diagnosis and treatment activities, OLDU behavior is inevitable in medical institutions. From the perspective of judicial and legislative practice, OLDU behavior may cause adverse consequences to medical institutions and physicians themselves, which to a certain extent will cause the following condition in medical practice: physicians tend to conservatively and strictly implement the instructions in order to avoid disputes and practice risks, rather than break through the instruction limits for disease diagnosis and treatment and patients’ benefits. From the perspective of contributing to the treatment of patients and promoting the development of medicine, OLDU has its rationality and it is inevitable; In order to avoid disputes between patients and medical institutions, and protect the rights and interests of patients [10], it is necessary to further standardize the management process of OLDU behavior in medical institutions.

It is suggested to standardize the management process of OLDU in medical institutions, establish internal institutional norms and approval procedures for OLDU, and conduct appropriateness review of physician’s prescriptions and orders [11], timely and regularly keep the records of OLDU plans, and establish a reporting mechanism for temporary medication. When adverse reactions occur, establish a monito**r**ing database for the adverse reactions of OLDU.

In view of the fact that most of the current OLDU information is reported in literature, and there is no unified information platform nationwide, it is suggested to establish a nationwide OLDU data system, including reporting, adverse reaction monitoring, etc., so as to promote specification formulation and improvement, providing a resource platform for rational supervision [12]. In view of the evidence-based medicine evidences which are more controversial between medical institutions and patients, relevant industry societies or academic organizations may organize assessment and release guidelines and bases related to drug use [13]. Strengthening the communication mechanism between physicians and pharmaceutical companies will help to prevent drug use risks and promote the development of clinical drug use.

## Conclusions

### Face up to the rationality of OLDU and protect physicians’ enthusiasm

Many cases of judicial precedents show that disputes about OLDU exist in practice. The newly revised Law of the People’s Republic of China on Physicians in 2022 explicitly allows OLDU in certain conditions from the legislative level, and although the judicial precedents in practice all occurred before the new Law of the People’s Republic of China on Physicians came into effect, it is also confirmed from the level of judicial practice that OLDU is not necessarily equivalent to an unreasonable prescription, and OLDU does not necessarily constitute infringement. In clinical practice, what really needs to be avoided is the damage caused to patients by drug misuse and other mistakes. OLDU behaviors that can benefit patients’ treatment should be encouraged and supported, but at the same time, the prerequisites should be strictly limited, that is, there is no effective or better treatment means, and there are evidence-based medicine evidences, such as clinical application guidelines, clinical diagnosis and treatment guidelines, etc. If the medical institution causes damage due to medical negligence, it shall still bear tort liability [14]. In addition, it is necessary to distinguish the process of experimental drugs and OLDU, and make it clear that OLDU is not a new drug research and development, but a reasonable extension of diagnosis and treatment based on clinical practice.

### Improve the signing of informed consent and respect patients’ right to know

According to the statistics of judicial cases, it can be seen that patients’ believing that medical institutions did not fulfill the obligation of informed consent accounts for a large proportion (57%). Article 29 of the Law of the People’s Republic of China on Physicians stipulates that the explicit informed consent of the patient is required for OLDU. According to Article 1219 of the Civil Code of China, medical personnel shall promptly explain to patients the medical risks, alternative medical solutions, etc., and obtain their explicit consent; if they cannot or should not explain to the patient, they shall explain to the patient’s close relatives and obtain their explicit consent. The Civil Code of China changes obtaining written consent in the original Tort Liability Law to obtaining explicit consent, so that the form of notification by medical institutions is no longer limited to written consent, instead the content of the notification should reach the full understanding of patients and their families. Although explicit informed consent is not the same as written consent, in the practice of diagnosis and treatment, written consent has evidentiary effect and helps to avoid or deal with disputes between patients and medical institutions. It is recommended that medical institutions formulate a specific informed consent template for OLDU and standardize relevant content of informing. The elements of the standardized template include: the explanation for OLDU, the evidence-based medicine evidences, possible medical risks, alternatives and the explanations of related reasons, and obtaining the confirming signature of the patient or their families. International studies indicate that when written information is provided to patients as part of the informed consent process, it should be fully understandable to the patient, regardless of their degree of literacy [15]. Firstly, it is recommended to use plain language, which means it has to be written in a clear and understandable language [16]. It is recommended to use short, simple and direct phrase with intelligible words (to use synonymous), not abusing of capital letters, not to place more than an idea by sentence when formulating written consent template for OLDU.

### Limitations of this study

In the process of studying judicial precedents related to OLDU, relevant information of some precedents is incomplete, and the extracted subjective and objective factors affecting the decision of the cases are not comprehensive enough. In addition, in China’s legal practice, there are not many cases of OLDU entering judicial procedure, which leads to the small sample size of this study. Further studies need to increase the sample size in the future, and try to expand to cases that have not yet entered judicial procedure, but do have disputes between hospitals and patients, so as to carry out a larger range of sampling and research.

## Data Availability

The datasets used and/or analysed during the current study available from the corresponding author on reasonable request.

## Abbreviations

OLDU: Off-label drug use.
